# Insights into age- and sickle-cell-disease- interaction using principal components analysis

**DOI:** 10.1186/1471-2326-6-3

**Published:** 2006-09-04

**Authors:** Mamta Sharma, Manju R Mamtani, Manik Amin, Tushar P Thakre, Smita Sharma, Amit Amin, Hemant Kulkarni

**Affiliations:** 1 Indira Gandhi Government Medical College, Nagpur, India; 2 Lata Medical Research Foundation, Nagpur, India; 3 Department of Integrative Physiology, University of North Texas Health Science Center, Fort Worth, TX, USA

## Abstract

**Background:**

In the context of sickle cell anemia, peripheral blood indexes provide key information that is also potentially influenced by age. Therefore, it is necessary to understand the extent and nature of interactions between sickle cell anemia and age, especially in situations where there is a high prevalence of sickle cell anemia.

**Methods:**

In a cross-sectional study of 374 subjects with varying hemoglobin S (HbS) status, we characterized the interaction between age and sickle hemoglobin using principal components analysis.

**Results:**

Factor analysis in subjects with hemoglobin AA identified three orthogonal factors – normal erythropoiesis, presence of thalassemia and the aggregability potential of the blood. These three factors were differentially associated with hemoglobin status. Age influenced the association of factors #2 and #3 with hemoglobin status.

**Conclusion:**

Our findings suggest that the interaction between age and hemoglobin status needs to be considered in both clinical and public health settings.

## Background

Sickle cell disease strongly influences the peripheral blood cell counts and red cell characteristics. Moreover, each of these parameters measured in the peripheral blood can be potentially instructive with regards the systemic alterations in sickle cell pathophysiology. However, since these peripheral blood indexes show complex correlations among each other [1,2] it is conceivable that they capture overlapping information about the disease process and pose difficulty for correctly interpreting the different and independent components that are affected in sickle cell pathology. One study [2] – using data from 825 homozygous sickle cell subjects – used principal components analysis and observed that the peripheral blood characteristics map on to three "factors" that represent hemolysis rate, presence of β-thalassemia and intra-erythrocytic hemoglobin polymerization. However, since this study used homozygous sickle cell subjects only, it did not permit an understanding of the extent to which the factors are altered in sickle cell anemia as compared to their normal configuration. Further, age is known to significantly affect the peripheral blood characteristics. Therefore, it becomes of practical as well as academic interest to explore if age differentially influences these factors.

## Methods

We conducted a cross-sectional study on 374 subjects – 196 (52.4%) with hemoglobin AA (HbAA), 97 (25.9%) with hemoglobin AS (HbAS) and 81 (21.7%) with hemoglobin SS (HbSS) pattern on cellulose acetate paper elecrophoresis at pH 8.4. The subjects were recruited from Departments of Pathology at two centers in India – Mahatma Gandhi Institute of Medical Sciences, Wardha (n = 216) and Indira Gandhi Government Medical College, Nagpur (n = 158). The characteristics of subjects recruited at the Wardha center have been described previously [3]. In this group of subjects ~69% were males and none of the studied blood parameters were significantly different across gender. In the Nagpur center, all the sickle cell subjects were males. Overall, our study sample was predominantly male (~82%). The two study centers were comparable with respect to the level of health care provided, socioeconomic status of the subjects, diagnostic protocol for sickle cell disease inclusive of hemoglobin electrophoresis and measurement of peripheral blood indexes on an automated cell counter, therapeutic protocol for sickle cell disease and the frequency of infectious disease observed in the sickle cell subjects (data not shown). There were 196 (52.4%) children (age<12 years), 71 (19.0%) adolescents (age 12 – <18 years) and 107 (28.6%) adults (age ≥ 18 years) in our study sample. The study protocol was approved by the Ethical Committee of the Indira Gandhi Government Medical College, Nagpur and the institutional review board of the Lata Medical Research Foundation.

We used principal components factor analysis (PCF) to gain insights into the pathophysiology of sickle cell anemia. This analysis permits the representation of each variable as a linear combination of the latent factors [4] as z_ij _= Σb_ik_f_kj _+ u_i_, where z represents the standardized value of the i^th ^variable for the j^th ^subject, b represents the factor loading of the i^th ^variable on the k^th ^factor, f represents the factor score of the k^th ^factor for the j^th ^subject and u represents the uniqueness of the i^th ^variable. For the purposes of our analyses, we used PCF on nine variables: red blood cell count (RBC), hemoglobin concentration (HB), packed cell volume (PCV), mean corpuscular volume (MCV), mean corpuscular hemoglobin (MCH), mean corpuscular hemoglobin concentration (MCHC), red cell distribution width (RDW), platelet count (PLT) and total leukocyte count (TLC).

Our overall analytical strategy was as follows: i) We used PCF only on the HbAA subjects. We undertook this step since we first needed to understand the principal components derived from normal subjects and second we needed to see if this derived factor structure is altered in subjects with the sickle cell gene – either in homozygous or heterozygous state. Hemoglobin AA genotype represents the normal non-mutated adult hemoglobin. Therefore this group of subjects served as the reference group for deriving the principal components. The PCF solution in this group of subjects was obtained using a criterion of a minimum eigenvalue of 1. Then we optimized the factor solution using varimax rotation. ii) Using the factor structure so obtained we estimated the factor scores on all subjects including the HbAS and HbSS. In other words we applied the factor structure of the HbAA subjects to all the subjects. iii) We then compared the factor scores for each identified factor across the HbAA, HbAS and HbSS groups as well as across the different age groups. Our decision to categorize age was based on the following reasons considered in unison: a) we wished to examine if particular clinically relevant age groups influenced the factor structure rather than whether age is a predictor of the factor scores; b) if age would have been used as a continuous variable then it would have constrained our analysis around an implicit assumption of a linear association between factor scores and age. However, as our age categorization indicates, we had no *a priori *reason for assuming this linear relationship; and c) we conducted analysis of variance using factor scores as the dependent variable and the sickle cell genotype and age as the predictors. The results of these analyses indicated (especially for the second factor score) that the model fit was better when age categories were used as the predictor. These results (data not shown) suggested that the assumption of linearity for our dataset was not valid. iv) We used analysis of variance (ANOVA) to understand the simultaneous and interacting influence of age and hemoglobin on the identified factor scores.

## Results

### Results of PCF analysis

**Figure 1:**
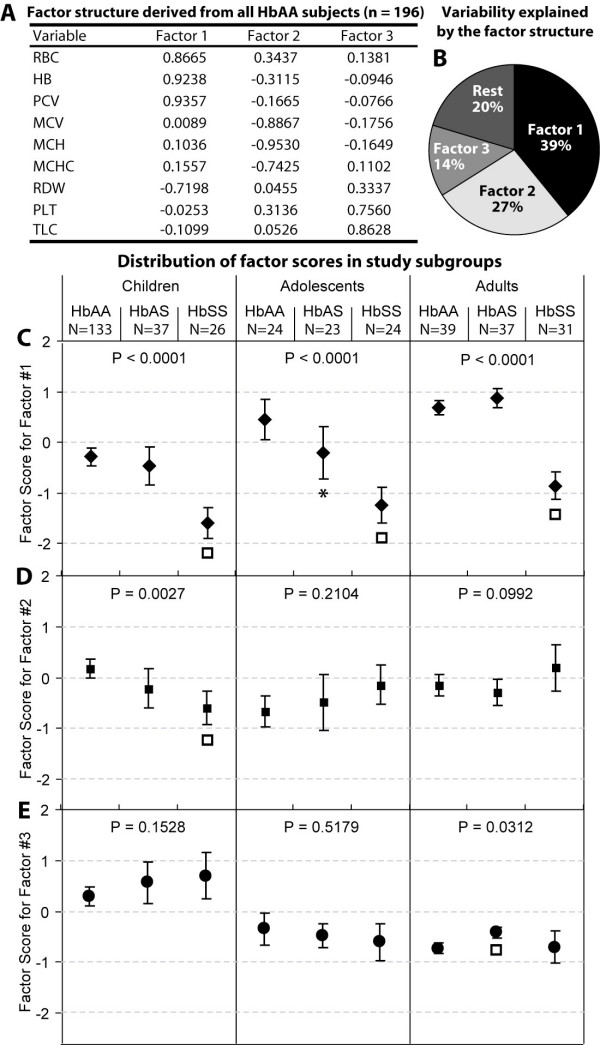
**Results of principal components factor analysis**. (A and B) The factor structure of HbAA study subjects and the variability explained by the retained factors. Numbers in panel A show the factor loadings. (C to E) Influence of age and hemoglobin status on the factor scores for the three factors shown in panel A. The point and 95% confidence intervals are depicted as markers (diamonds for factor #1, filled squares for factor #2 and circles for factor #3) and error bars, respectively. The analysis is shown for all combinations of age categories and hemoglobin status. For each age category and factor the influence of hemoglobin was assessed by ANOVA (p values shown at the top). Also, the factor scores for HbAS and HbSS subjects were compared with those of HbAA subjects for each combination using Mann-Whitney test. *, Bonferroni-corrected p < 0.05; ϒ, Bonferroni-corrected p < 0.01.

The results of PCF in the HbAA subjects are shown in Figure 1A and 1B. Our analysis identified three orthogonal factors. On the first factor, RBC, HB and PCV loaded strongly positively while RDW loaded strongly negatively suggesting that this factor represented normal erythropoiesis [5]. On the second factor, MCV, MCH and MCHC loaded strongly negatively raising the possibility that this factor perhaps related to iron deficiency anemia and/or thalassemia – two forms of anemia common in the study setting. However, the fact that the RBC loaded positively while RDW only minimally loaded on this factor indicated [6] that factor was less likely to be iron deficiency anemia and we thus named this factor as the thalassemia factor. On the third factor, PLT and TLC loaded strongly positively while RDW loaded moderately positively pointing towards the possibility that, in the context of sickle cell anemia, this factor indicated the aggregability potential of the blood since red cell anisocytosis, leukocytosis and thrombocytosis are known to increase the blood viscosity [7] as well as influence the vascular endothelial reactivity [8]. Together these factors explained ~80% of the variability in the nine variables studied.

### Assessment of the factor scores

When we examined the factor scores across the age categories and hemoglobin status of the study subjects (Figure 1C to 1E), we noted several interesting observations. First, the normal erythropoiesis factor scored significantly low in the HbSS subjects at all ages. Second, the scores of factor #1 demonstrated a consistent trend across hemoglobin status (HbAA>HbAS>HbSS) at all ages. Third, the thalassemia factor scores were significantly reduced in HbSS subjects in children but not at higher ages indicating that the coexistence of thalassemia and sickle cell anemia is more likely at higher ages. Indeed, when we conducted ANOVA with an interaction term we observed that the interaction between age and hemoglobin status was highly significant (F = 4.17, p = 0.0026) while neither age nor hemoglobin status significantly predicted the factor score for the thalassemia factor. Fourth, the increase in the aggregability potential factor was increased significantly only in HbAS subjects in adults although a trend (p = 0.15) to an increased aggregability potential was also seen in children.

## Discussion

By its very nature, explorative factor analysis is essentially a speculative tool. The factors identified and named in a factor analysis always need to be considered in the light of the existing knowledge. Consequently, it can not be claimed that the factor structure obtained within a sample is completely generalizable. Also, since our sample was not representative of females, we can not comment about the influence of gender on our inference. Nevertheless, what we attempted in the present study is to examine if factor structure is different across subjects with differing hemoglobin status and age categories and we found important differences.

Our results have at least two implications. First, the coexistence of thalassemia and sickle cell anemia seems to be age-related. Again, in the absence of knowledge about the hemoglobin A_2 _concentration in the study subjects it will not be possible to emphatically conclude about the coexistence of these two conditions. However, our finding corroborates the fact that the morbidity and mortality of both thalassemia and sickle cell anemia is more in children [9] making the detection of coexistence difficult in a study like ours. By contrast, it can be argued that adults who have survived both these challenges in childhood are likely to demonstrate a higher prevalence of these two disorders simultaneously in populations where both the disorders are common. Screening programs for these two conditions need to be made aware of this possibility. Second, the fact that factor #3 showed a higher score in sickle cell anemia indicates that myeloid hyperplasia in addition to erythroid hyperplasia can occur in sickle cell disease especially at higher age. This argument concurs with the several recent findings that report thrombocytosis as well as leukocytosis in sickle cell disease [3,10].

## Conclusion

On a wider scale, our findings demonstrate the need to consider age in correctly interpreting peripheral blood picture in sickle cell anemia.

## Competing interests

The author(s) declare that they have no competing interests.

## Authors' contributions

MS, MRM and HK conceptualized the study. MS, MA, SS and AA participated in data collection. MRM and HK conducted the statistical analyses. HK wrote the initial draft of the manuscript. TPT critically reviewed and revised the manuscript. All authors read and approved the final manuscript.

## Pre-publication history

The pre-publication history for this paper can be accessed here:


